# Rapid Recovery of Visual Acuity after Lumboperitoneal Shunt Operation in Malignant Idiopathic Intracranial Hypertension

**DOI:** 10.1155/2011/405838

**Published:** 2011-10-20

**Authors:** Levent Sinan Bir, Eylem Degirmenci, Cagdas Erdogan, Selma Bilgin, Erdal Coşkun

**Affiliations:** ^1^Neurology Department, Faculty of Medicine, Pamukkale University, 90-20020 Denizli, Turkey; ^2^Neurosurgery Department, Faculty of Medicine, Pamukkale University, 90-20020 Denizli, Turkey

## Abstract

*Background*. Idiopathic intracranial hypertension can cause rapid deterioration of visual acuity in some severe cases, and these cases are usually thought to have “malignant” form of this disease. *Case*. In this paper, we report on a 16-year-old girl who is a typical example for malignant idiopathic intracranial hypertension with a rapid recovery of visual acuity after lumboperitoneal shunt operation. *Observations and Conclusions*. Malignant form of idiopathic intracranial hypertension must be kept in mind in selected patients to avoid irreversible visual loss.

## 1. Introduction

Idiopathic intracranial hypertension (IIH) is a syndrome of increased intracranial pressure without hydrocephalus or mass lesion and with normal cerebrospinal fluid (CSF) composition [1]. The course of IIH is generally favorable, but rarely severe visual loss may occur progressively, as the result of chronic papilledema in the late stage of the disease [2]. In very rare situations, severe visual loss may occur as an acute event, defining the so-called “malignant” IIH, and final visual outcome in these patients is variable [3]. We report a case of IHH presenting with acute, rapidly progressive visual loss that showed rapid recovery of after lumboperitoneal shunt operation. 

## 2. Case Report

A 16-year-old girl referred to our hospital with complaint of a 9-day history of severe, pulsatile, and continuous headache with nausea and vomiting. We learned that blurred vision which was more prominent in her left eye had started 2 days after the beginning of headache, and she was brought to a hospital. She was found to have bilateral disk edema, and a computerized tomography (CT) scan of the brain was performed and showed normal findings. Lumbar puncture (LP) was performed and the opening pressure was found high. After performing the cranial, orbital, and cervical magnetic resonance imagings (MRIs), which were unremarkable, and normal cerebral spinal fluid analysis, she was referred to our hospital. 

**Figure 1:**
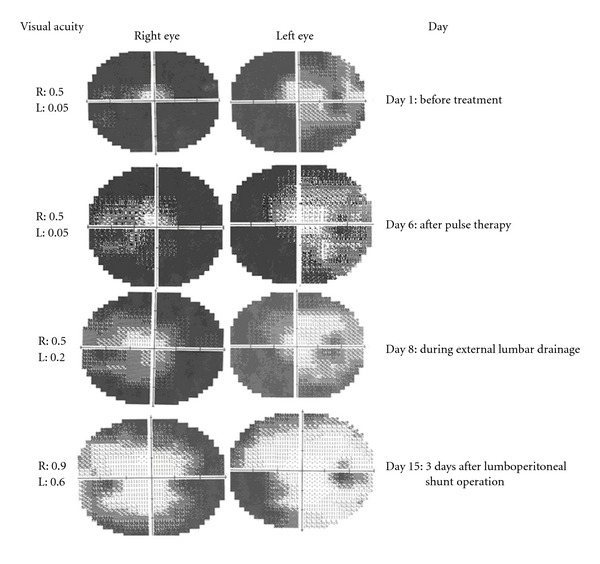
The results of patient's visual field examination and corrected visual acuity according to the day of hospitalization.

On admission, her headache and vomiting was going on, and we learned that her blurred vision got worse during this period. Bilaterally papilledema was seen on funduscopic examination. Extraocular movements were intact, and there was no ptosis or nystagmus. Her best corrected visual acuity was 0.5 in the right eye and 0.005 in the left eye, and we found severe concentric visual field constriction in her perimetric visual field examination. Other neurological examination findings were normal. In her physical examination BMI was within normal limits (22 kg/m^2^), and signs of sinusitis were determined. Her medical history was unremarkable for IIH. Her basic metabolic panel values, thyroid function test, and parathormone were normal except white complete blood count indicating thalassemia intermedia. MR angiography and venography that were performed to exclude the possibility of a cerebral venous thrombosis were normal. Laboratory tests for the etiology of IIH (ANA, Anti-DNA, ANCA, ANA profile, Protein C, S, antithrombin III resistance, activated protein C resistance, Lyme screen, Brucella tests, and VDRL) were all normal. A repeated LP showed an opening pressure of 380 mmHg with normal CSF analysis. After that, acetazolamide therapy (1000 mg per day, po) and methylprednisolone pulse therapy (1 g per day, iv) were given immediately, but her visual acuity did not show any improvement. Her best corrected visual acuity was found 0.5 at the right side and 0.05 at the left eye. These treatments showed only a minor affect in her headache, and an emergent lumbar drainage was performed at the sixth day of the hospitalization. After starting external lumbar drainage, the patient's visual acuity and visual field examination showed improvement (best-corrected visual acuity; 0.5 in the right and 0.2 in the left eye). With these results, a permanent lumboperitoneal shunt was planned, and during preoperative preparations external lumbar drainage was stopped. After 36 hours, a right peripheral facial palsy was observed, and her external lumbar drainage was replaced with a permanent lumboperitoneal shunt emergently. This treatment stabilized visual symptoms and was followed by a rapid improvement of visual acuity after operation. Visual acuity was found 0.9 at the right side and 0.6 at the left side after the operation. The results of patient's visual field examinations and corrected visual acuity according to the day of hospitalization are shown in Figure 1. 

## 3. Discussion

In general practice, it is well known that visual complaints are typically absent at presentation of IIH, but affected visual acuity may occur in the late course of the disease; in particular, if there is longstanding papilledema. However, the acuity may deteriorate rapidly in severe cases of IIH [4, 5]. These cases are usually thought to have “malignant” or “fulminant” IIH, and our case is a typical example for malignant IHH. Visual symptoms had nearly started concurrently with headache in our case and deteriorated in a few days. Such patients as our case who generally have marked visual field loss or decreased central vision at presentation require urgent treatments like repeated lumbar punctures, lumbar drainage, and intravenous steroids [3]. We first started diuretic and steroid therapy, but, because of failed medical therapy and intractable headache and with the aim of preventing visual acuity, we planned urgent surgical treatment.

Two goals must be considered when managing patients with idiopathic intracranial hypertension: prevention of visual loss and resolution of symptoms. Presence of severe visual loss (20/40 or worse) in one or both eyes at time of initial examination is one of the indications for surgery [1]. Our case's visual acuity was 20/100 in the right eye and 5/100 in the left eye on admission. It is estimated that up to 17 percent of patients with IIH will progress to develop permanent visual loss [3]. The final visual outcome in fulminant IIH is variable but, up to half of the patients, could become totally blind [6]. 

Mensah et al. [3] reported that patients with marked visual field constriction and severe visual acuity loss at the time of onset of the IIH showed only partial response to therapy with diuretics and steroids and these patients have suffered clinically malignant course with rapid progression to near blindness. In light of the foregoing clinical experiences, we planned an urgent operation to avoid blindness. 

Malomo et al. [7] reported a case with a 4-week history of persistent of IHH with visual loss and deafness that had a good response to the nonoperative treatments; however, Kidron and Pomeranz [8] reported two cases with malignant IHH and one of these patients underwent lumboperitoneal shunt operation. In fact, it is not well known about malignant form of IHH, and there is no obvious explanation of the disastrous outcome in these patients, but, in agreement with occasional reports in the literature, it is strongly suggested not to squander time so as to protect the patient's visual acuity. 

As a result, we believe that such cases, as presenting case, need to be recognized as the malignant end of the spectrum of IHH, and that their management requires a neurological emergency. Although rare, keeping this malignant form IHH in mind and choosing the right acute treatment option might cause full recovery of visual acuity in a nearly total blind patient.

##  Disclousure

The authors alone are responsible for the content and writing of the paper.
